# Self-Assembled 3D Actuator Using the Resilience of an Elastomeric Material

**DOI:** 10.3389/frobt.2019.00152

**Published:** 2020-01-15

**Authors:** Naoki Hashimoto, Hiroki Shigemune, Ayato Minaminosono, Shingo Maeda, Hideyuki Sawada

**Affiliations:** ^1^Department of Applied Physics, Waseda University, Tokyo, Japan; ^2^Division of Mechanical Engineering, Shibaura Institute of Technology, Tokyo, Japan

**Keywords:** soft robotics, dielectric elastomer actuator, self-folding method, self-assembled actuator, gripper

## Abstract

Self-folding technologies have been studied by many researchers for applications to various engineering fields. Most of the self-folding methods that use the physical properties of materials require complex preparation, and usually take time to complete. In order to solve these problems, we focus on the elasticity of a material, and propose a model for forming a 3D structure using its characteristics. Our proposed model achieves high-speed and high-precision self-folding with a simple structure, by attaching rigid frames to a stretchable elastomer. The self-folded structure is applied to introduce a self-assembled actuator by exploiting a dielectric elastomer actuator (DEA). We develop the self-assembled actuator driven with the voltage application by attaching stretchable electrodes on the both side of the elastomer. We attempt several experiments to investigate the basic characteristics of the actuator. We also propose an application of the self-assembled actuator as a gripper based on the experimental results. The gripper has three joints with the angle of 120°, and successfully grabs objects by switching the voltage.

## Introduction

A technology of “origami” that creates a 3D structure by folding a flat sheet such as a paper and a metal plate has been studied by many researchers. In recent years, new techniques related to origami have been widely studied in the field of engineering. By folding a large sheet into a small piece, the sheet object is hardly carried to different places to be extended into complex 3D structures. For example, space structures (Torisaka et al., 2016), solar cell arrays (Natori et al., 2013), protein origami (Dobson, 2003), and folding of insect wings (Saito et al., 2017) are the successful applications of origami studies.

On the other hand, the research on self-folding mechanism has been actively conducted by utilizing physical properties of materials. Examples of such prior studies are found in heat-shrinkable polymer (Liu et al., 2012, 2017; Felton et al., 2014) and paper (Shigemune et al., 2016, 2017).

We focus on an elastomeric material, and introduce a self-folding mechanism that utilizes the resilience of the material. Elastomers have several properties such as being capable of large deformations of several hundreds percent, superior processability, and high compatibility with living organisms in terms of stretchablity. Furthermore, it can also be applied to an actuator by designing conductive electrode.

Dielectric Elastomer Minimum Energy Structures (DEMES) (Araromi et al., 2015) is a device that forms a 3D structure, in which the elastic energy of the elastomer and the folding energy of the flexible frame are minimized. This structure shows large deformation by the voltage application, and enables the manufacture of lightweight and flexible actuators. However, the research mainly focuses on obtaining larger displacements with the Dielectric Elastomer Actuator, and it has difficulty to form precise self-folded structures. Mintchev et al. introduced a 3D structure formation method by combining a rigid frame with a flexible elastomeric material (Mintchev et al., 2017). This structure displays both high load bearing and high resilience characteristics. The research focuses on reducing the impact on the device from the outside, and is not intended for the use as an actuator.

In this research, we propose a self-folding method using the characteristics of the elastomeric material. Our proposed mechanism uses the resilience of the elastomer for assembly, therefore the total system can be untethered. This assembly does not require external stimuli, and the assembling time is much shorter than the other methods. The self-folded structure can naturally introduce a self-assembled electric actuator called a Dielectric Elastomer Actuator (DEA), by applying stretchable electrodes to the elastomer. We demonstrate the potential ability of the self-assembled actuator by applying it as a gripper.

## Mechanism

### Self-Folding Model Using an Elastomer and Rigid Frames

**Figure 1:**
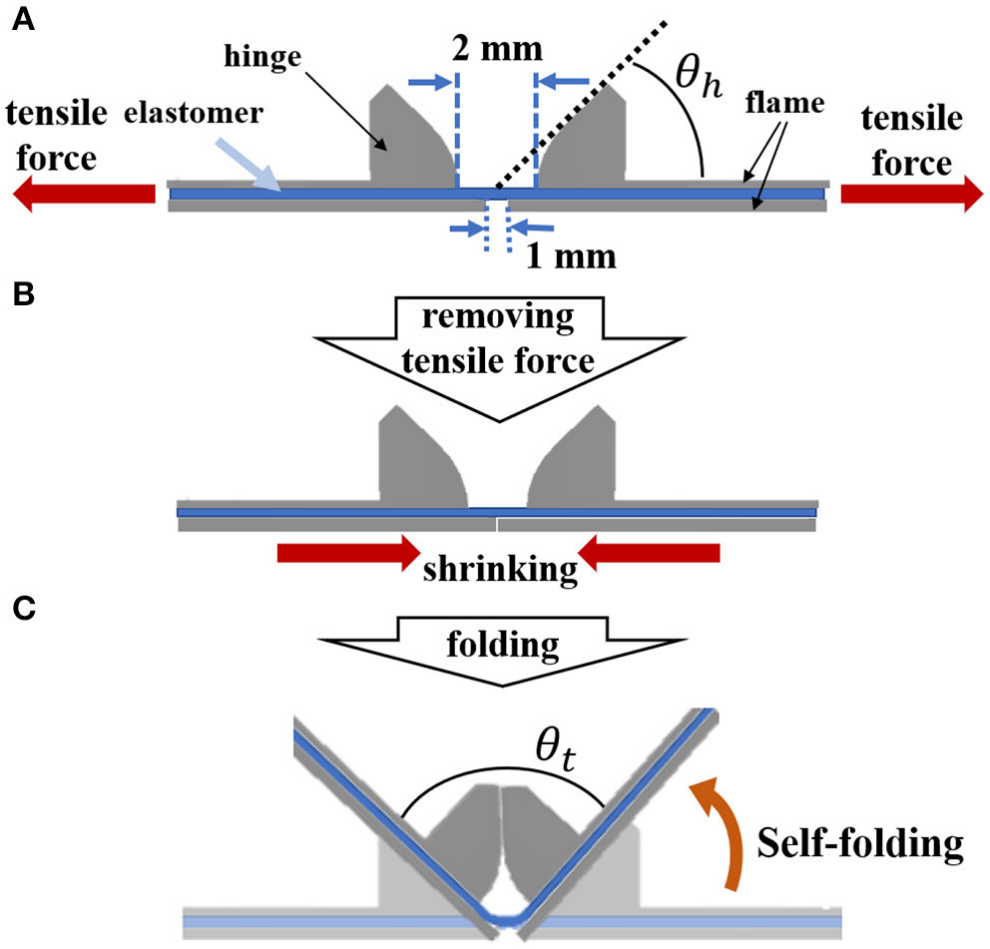
Mechanism of forming a 3D structure. **(A)** Attach two hinged and non-hinged frames to both sides of the pre-stretched elastomer. **(B)** When the tensile force is removed, the elastomer shrinks and the non-hinge frames collide, which has a shorter distance between the frames. **(C)** The contraction force of the elastomer produces a 3D structure, and the folding angle θ_*t*_ is determined by the angle θ_*h*_ of the hinge.

We propose a model that automatically forms a 3D structure using the resilience of an elastomer by pasting multiple rigid frames on both sides of a pre-stretched elastomer, as shown in Figure 1. First, we prepare a pre-strained elastomer using a rigid frame, and attach two hinged frames on a folding side and two frames without a hinge on the other side of the folding side. At this time, the distance between the hinge frames on the folding side is made wider than the frames on the other side (Figure 1A). Next, when the tensile force applied to the elastomer is removed by taking off from the rigid frame, the restoring force of the elastomer causes contraction, and the frames with shorter intervals come in contact with each other to restrict folding toward the side (Figure 1B). Then, the sheet bends toward the hinge frames with the remained gap (Figure 1C). The folding direction can be controlled in this way.

A method similar to the proposed method, in which hard frames are spaced apart and self-folding using the thermal contraction of the shape memory polymer, has already been studied (Tolley et al., 2014). In this method, the folding angle is controlled by adjusting the distance between the frames fixed on both sides of the shape memory polymer. On the other hand, the folding angle in our method can be adjusted by the different approach. The minimum bending angle can be designed in advance by attaching a hinge to the frame to constrain the bending angle. Furthermore, our method uses the shrinkage of the elastomer, and the time required for structure formation is much shorter than the thermal effect.

As shown in Figure 1, a target folding angle θ_*t*_ after self-folding can be arbitrarily determined by the size of the angle θ_*h*_ of the hinge portion of the frame, which can be expressed by Equation (1).

(1) $$\begin{array}{l} \begin{array}{c} \theta_{t}=\;2\theta_{h},\; \end{array} \end{array}$$

When assembling a 3D structure using two frames with the same hinge angle θ_*h*_, the θ_*h*_ should be set to half the θ_*t*_.

### Activation of Dielectric Elastomer Actuator

**Figure 2:**
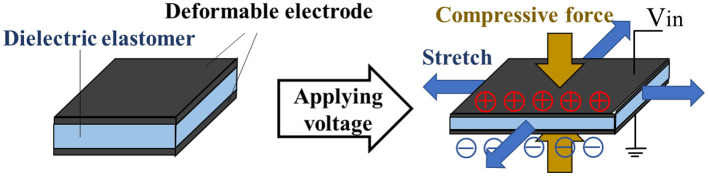
Structure and principle of DEA. When voltage is applied to the elastomer having deformable electrodes attached to both sides, it extends in the horizontal direction.

An elastomer film is sandwiched by the frames to self-fold. A self-assembled actuator is fabricated by applying carbon nanotube (CNT) electrodes on both sides of the elastomer. In order to drive the 3D structure introduced in section self-folding model using an elastomer and rigid frames, we employ a Dielectric Elastomer Actuator (DEA). Figure 2 shows the structure of DEA, in which a dielectric elastomer is sandwiched by stretchable electrodes to work as a capacitor. DEA has advantages of its simple and lightweight structures, being compatible with low energy consumption and fast response time (Plante and Dubowsky, 2007). In order to greatly deform the electrode part of DEA, the application of pre-strain is necessary. Pelrine showed that the maximum amount of deformation in the area of the elastomer when voltage is applied increases when the acrylic elastomer is properly pre-strained (Pelrine et al., 2000). DEA is applied for a versatile gripper (Shintake et al., 2016), a deformable motor (Minaminosono et al., 2019), and a balloon speaker (Hosoya et al., 2019) by exploiting its characteristics. Conversely, there are also studies that use DEA without pre-stretching the elastomer (Duduta et al., 2016). In such cases, however, it is necessary to reduce the thickness of the electrode sufficiently or to form a multilayer structure.

In this study, we use CNT powder as the stretchable electrodes. The application of voltage to the electrodes generates electrostatic attraction between the electrodes, which results in the elastomer being compressed and deformed in the horizontal direction. The compressive force *p*(*N*/*m*^2^) due to this electrostatic attraction can be expressed by Equation (2) (Wissler and Mazza, 2007).

(2) $$\begin{array}{l} \begin{array}{c} p=\;\varepsilon_{0}\varepsilon_{r}\frac{V^{2}}{z^{2}},\;\\ \; \end{array} \end{array}$$

where, ε_0_ is the permittivity of vacuum$\left(\varepsilon_{0}=8.854\times 10^{-12}\text{F}/\text{m}\right)$, ε_*r*_ is the relative permittivity of elastomer, *V* is the applied voltage, and *z* is the distance between electrodes. According to Equation (2), by using an elastomer with a high dielectric constant and a thin film thickness as a material of DEA, larger compressive force and large deformation can be generated. The compressive force also increases in proportion to the square of the applied voltage (O'Hallorana et al., 2008).

**Figure 3:**
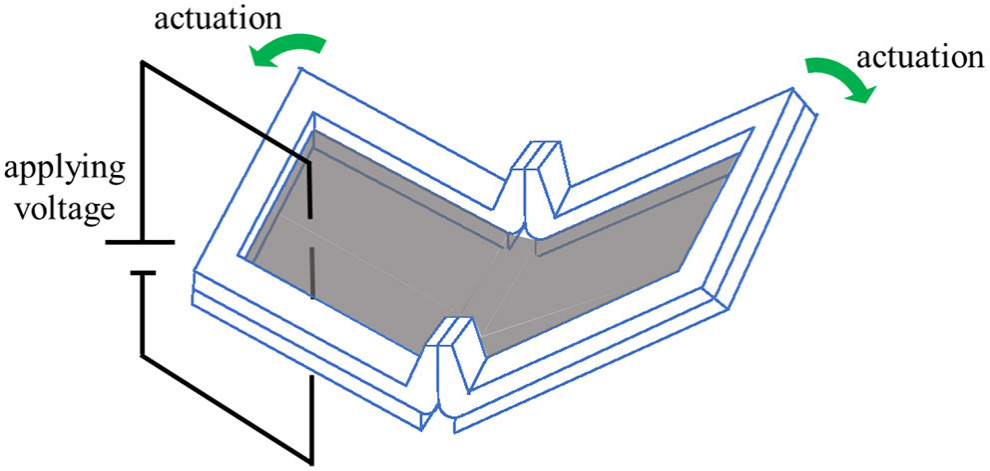
Appearance of self-assembled actuator and mechanism of actuation. The gray area represents an area which the electrode is attached. The electrode is attached to both sides of the elastomer. When a voltage is applied to the electrodes, the actuator is driven in the opening direction.

The self-assembled actuator not only automatically forms a 3D structure, but also maintains the pre-strain of the elastomer to be activated as the DEA. The pre-strained elastomer extends by applying voltage toward the applied CNT electrodes. Figure 3 shows a self-assembled actuator fabricated by attaching electrodes to a 3D structure fabricated by self-folding. When a voltage is applied to the electrode, the expansion of DEA is converted toward displacement in the direction to open the hinge.

## Fabrication

### Frame

**Figure 4:**
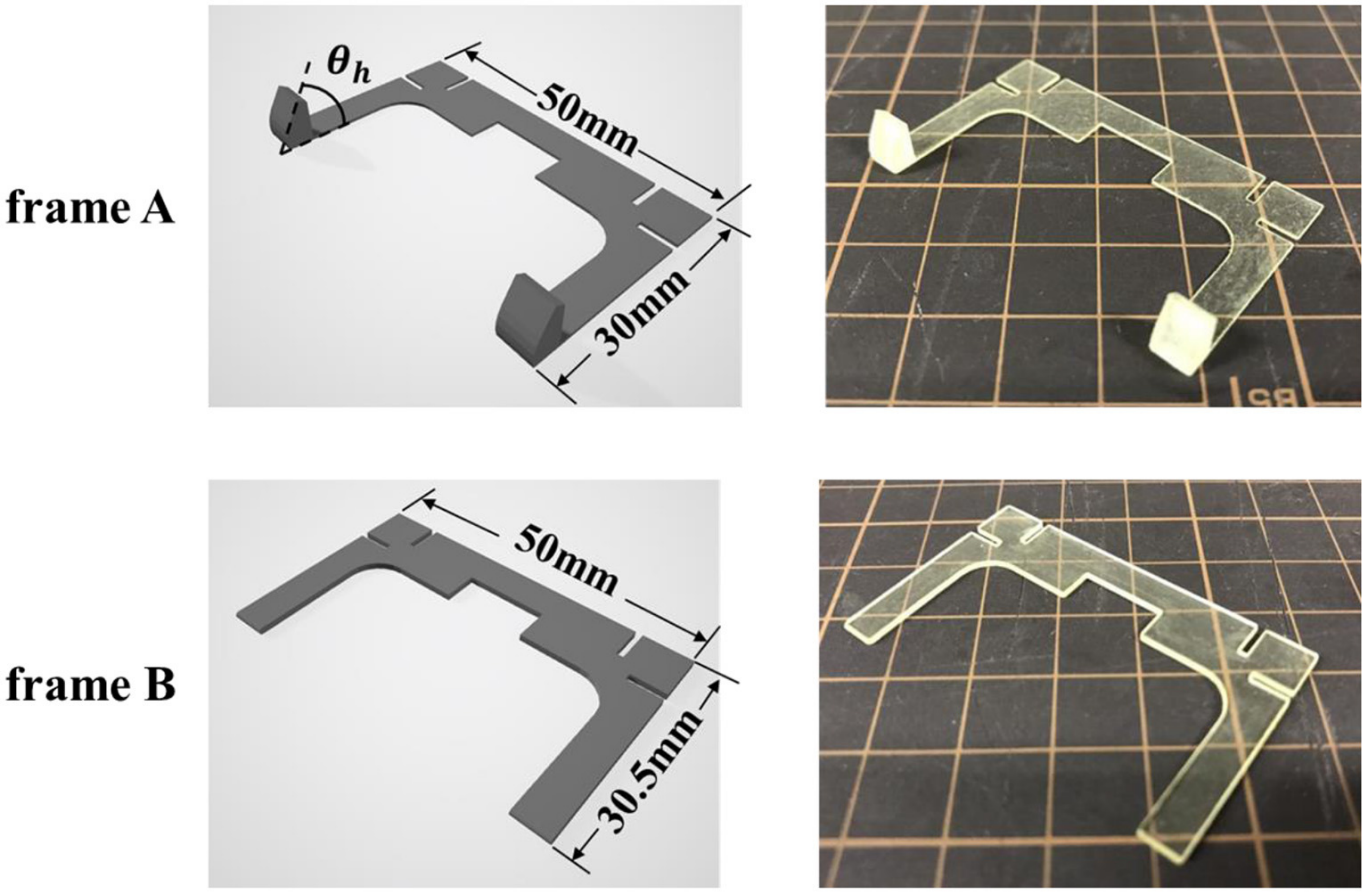
Frame data designed in CAD software and actual printed frame. In order to realize the self-folding described in Figure 1, the length of the short side of frame B was designed to be 0.5 mm longer than that of frame A. The thickness of each frame is 0.5 mm. Here, the case of θ_*h*_ = 60 is shown as an example of the frame A.

Two types of frames were designed to realize self-folding with the proposed model. A 3D-CAD (Auto CAD) was employed for designing, and the fabrication was conducted by a 3D printer (AGILISTA-3200; KEYENCE, Tokyo, Japan). Figure 4 shows the design data and the photos of the frames. When designing the frame, the following two points were taken into consideration. The first point is to attach a hinge to determine the folding angle. The hinges of the frame A limit the folding due to the shrinkage of the elastomer by colliding with each other, and contribute to the determination of the folding angle. The length of the side of frame B was designed to be slightly longer than that of frame A so that it would collide first when the elastomer contracted. As a result, the entire structure folds in the direction of frame A. In this way, the frame A plays a role in determining the folding angle, and the frame B plays a role in determining the direction of folding. The second point is to fix the pre-stretch of the elastomer at the part where the electrode is applied to make DEA. As explained in section activation of dielectric elastomer actuator, a pre-strained DEA is greatly deformed. In our preliminary experiment, we discovered that 300% pre-strain should be uniformly applied for the best performance by using an acrylic elastomer (VHB 4905-J; 3M Company, Saint Paul, Minnesota, United States).

### 3D Structure Fabricated by Self-Folding

**Figure 5:**
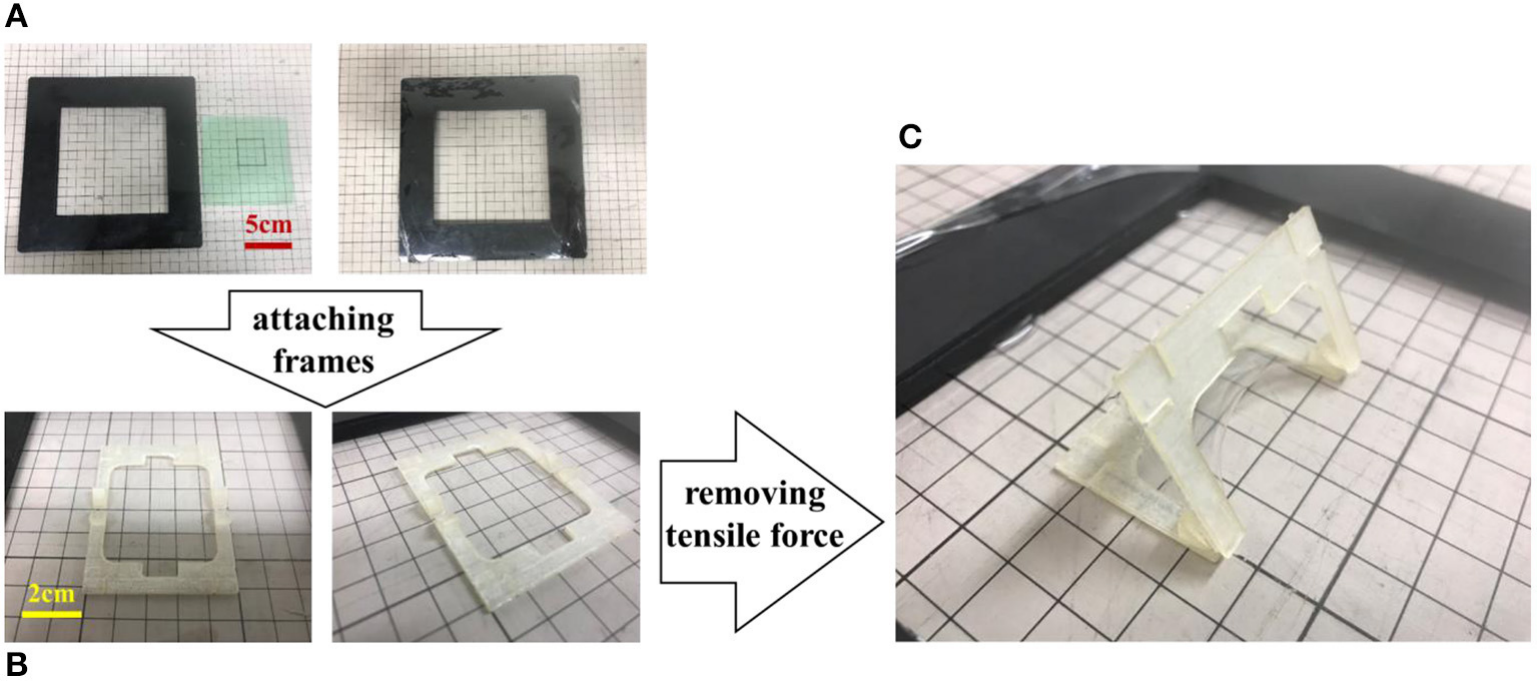
Process of fabricating a self-folding prototype. **(A)** Elastomer is fixed with 300% pre-strain. **(B)** Frames are attached to both sides of the elastomer. **(C)** A 3D structure is spontaneously formed using the resilience of the elastomer. Self-folding is completed in about 1 s.

Figure 5 shows the fabrication process of a 3D structure using our proposed mechanism. First, we prepare a 300% pre-stretched elastomer, and attach two frames (frames A and B) to each side of the elastomer (Figure 5A). The distance between frames B should be shorter than the distance between frames A. In this experiment, the interval between frames A was set to 2 mm, and that between frames B was set to 1 mm (Figure 5B). By removing the elastomer from the frame, a 3D structure is spontaneously formed using the resilience of the elastomer (Figure 5C). Self-folding is completed in about 1 second after the tensile force of the elastomer is removed. A video recording the process of creating the self-folded structure is attached as Supplementary Material.

**Figure 6:**
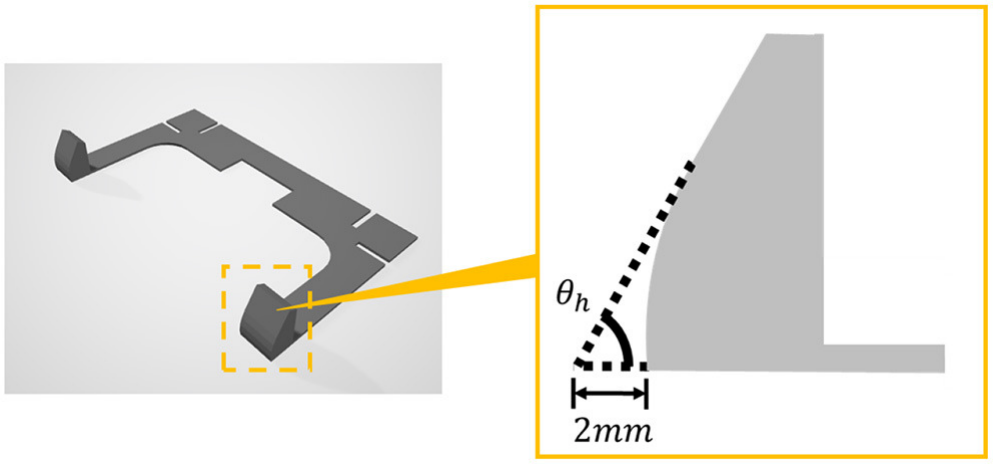
The structure of Frame A and enlarged view of hinge part. Hinge part is rounded as shown in the right figure so as not to impede the drive of the structure by applying voltage.

Figure 6 shows a side view of the hinge part of frame A. The surface where the hinges contact with each other is flat, and the surface close to the bottom is partly curved as shown in the figure. In this way, Frame A not only determines the folding angle, but also plays a role in helping the smooth operation when the voltage is applied to the self-assembled actuator. All four types of frames A with different angles θ_*h*_ are designed so that the distance from the intersection of the extension lines of the flat part of the hinge and the bottom to the bottom of the frame becomes 2 mm.

**Figure 7:**
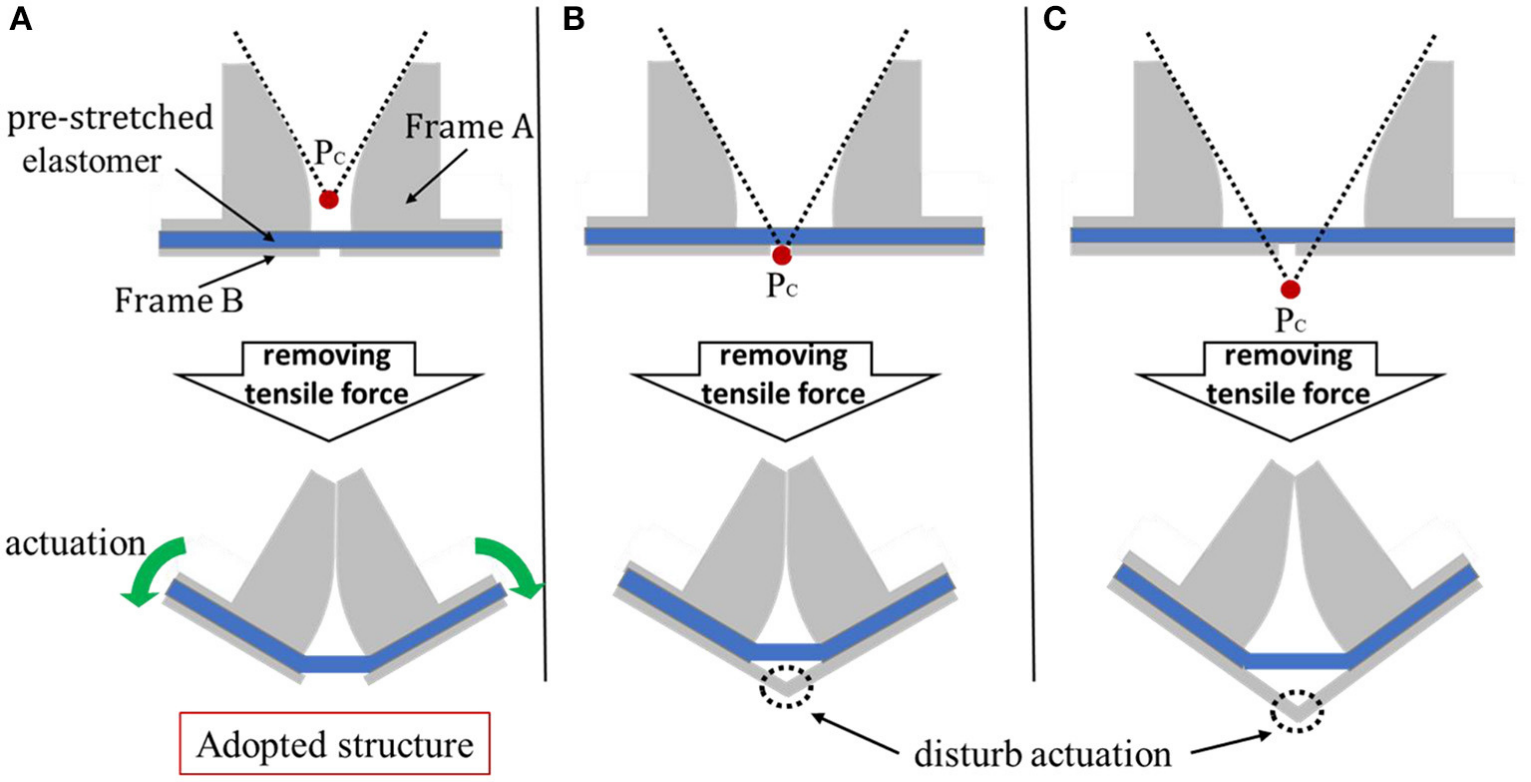
Three examples of relationship between gaps and self-folding between frames A. In this research, in order to optimize the operation of the actuator, we designed the distance between frames A to be 2 mm and that of B to be 1 mm as shown in **(A)**. **(A)** The case where point Pc is above the plane where Frame B exists. This structure was adopted in this research. **(B)** The case where the point Pc is on the plane where Frame B exists. In this state, the actuator does not operate when voltage is applied. **(C)** The case where point Pc is below the plane where Frame B exists. As in case B, the actuator does not operate.

Figure 7 shows the difference in bending when the gap between frames A is changed. Here, the point P_C_ is the point where the extension lines of the planar parts of the hinges of the two Frame A intersect. Figure 7A shows the case where the point P_C_ is above the plane where Frame B exists. In this state, when the tensile force applied to the elastomer is removed, self-folding occurs due to the contraction of the elastomer, and the frame A is fixed in a state where the plane parts are in contact with each other and the frame B is separated. Figure 7B shows the case where the point P_C_ is on the plane where Frame B exists. When the tensile force is removed, the frame A is fixed in a state where the plane parts are in contact with each other and the frames B are also in contact with each other. Figure 7C shows the case where point P_C_ is below the plane where Frame B exists. When the tensile force is removed, the frames A touch each other with a line instead of a face as shown in the figure, and the frames B are fixed in contact with each other. As shown in Figure 3, when the voltage is applied to the self-assembled actuator, the actuator is driven to open. When the frame B is in the state (a) where the frames B are separated from each other, the operation is not disturbed by the frame and it can be said that the operation works best. When the interval between frames A is 2 mm and the interval between frames B is 1 mm, the state is as shown in Figure 7A.

### Self-Assembled Actuator

**Figure 8:**
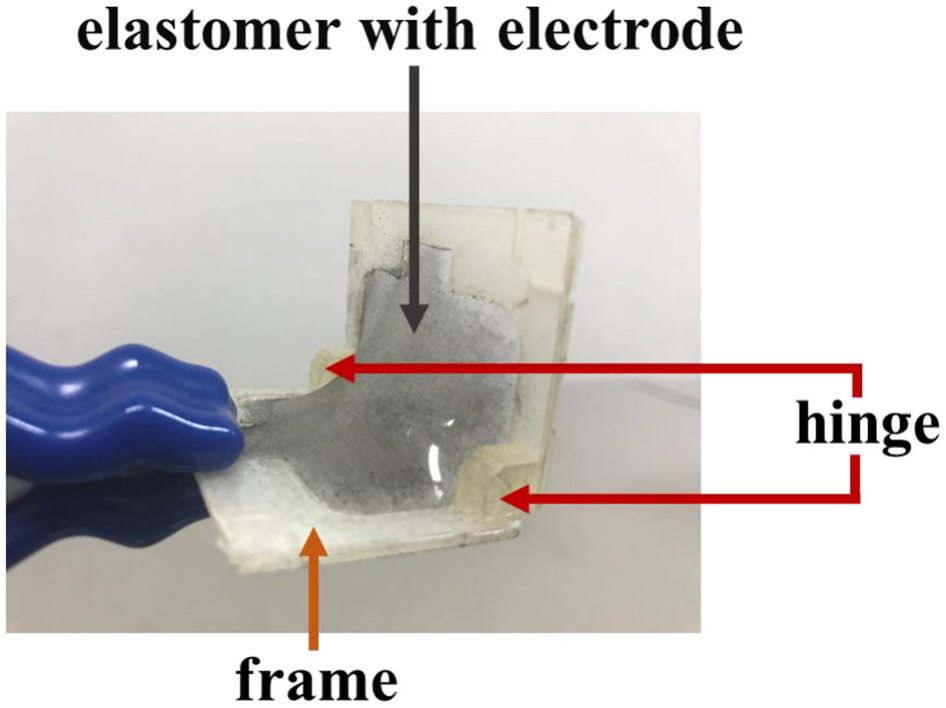
Appearance of the self-assembled actuator.

A self-assembled actuator is fabricated by attaching electrodes to the fabricated 3D structure. The actuator works by applying voltage to the electrodes as shown in Figure 8. The actuator displaces to increase folding angle. In this study, the self-assembled actuator is fabricated by brushing multi-walled carbon nanotubes (MWCNTs) on the elastomer part of the 3D structure as proposed in our previous study (Shigemune et al., 2018).

## Experiments

### Accuracy of Self-Folded Angle Based on a Designed Angle θ_*d*_

#### Experimental Condition

**Figure 9:**
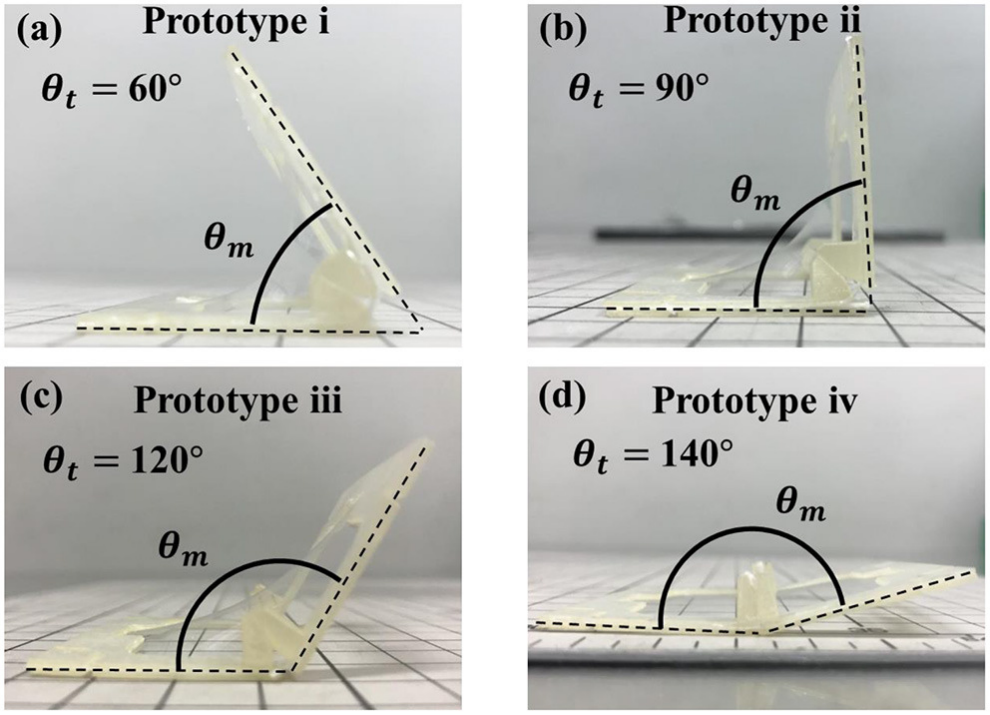
Four prototypes with different target angles θ_*t*_ to measure self-folding angles θ_*m*_. **(a)** The state of target angle θ_*t*_ = 60°. **(b)** The state of target angle θ_*t*_ = 90°. **(c)** The state of target angle θ_*t*_ = 120°. **(d)** The state of target angle θ_*t*_ = 140°.

In order to verify the accuracy of the proposed model, we conducted an experiment to investigate the relationship between the hinge angle θ_*h*_ and the actual folding angle θ_*m*_. Figure 9 shows four prototypes of 3D structure achieved by self-folding. The four prototypes i to iv with a target angle θ_*t*_ of 60°, 90°, 120°, and 140° were designed and fabricated, by using two frames A with the hinge angle θ_*h*_ of 30°, 45°, 60°, and 70°, respectively. We fabricated five samples for each prototype i to iv to measure the self-folded angle θ_*m*_ shown in Figure 9. We measured the angles by using the image analysis software (Kinovea) referring to the photos taken parallel to the ground.

### Results and Discussions

Table 1 shows the results of the experiment. The self-folded angles had small difference within 2 degrees from the initial designs i, ii, and iii. Prototype iv showed a larger difference of 14.08° to the opening direction. The standard deviation became larger when the self-folded angle was obtuse.

**Table 1:** Measurement results of self-folded angle θ_*m*_.

	**i (60°)**	**ii (90°)**	**iii (120°)**	**iv (140°)**
Average of θ_*m*_ (degree)	59.09	88.91	121.89	154.08
Error from θ_*t*_ (degree)	−0.91	−1.09	1.89	14.08
Standard deviation	0.85	0.87	5.49	5.78

From the results, we found that the self-folded angle is highly affected by the gravity caused by the weight of the frames and the elastomer. The self-folded angle is determined by the balance between the tension of the elastomer and the gravity applied to the elastomer.

**Figure 10:**
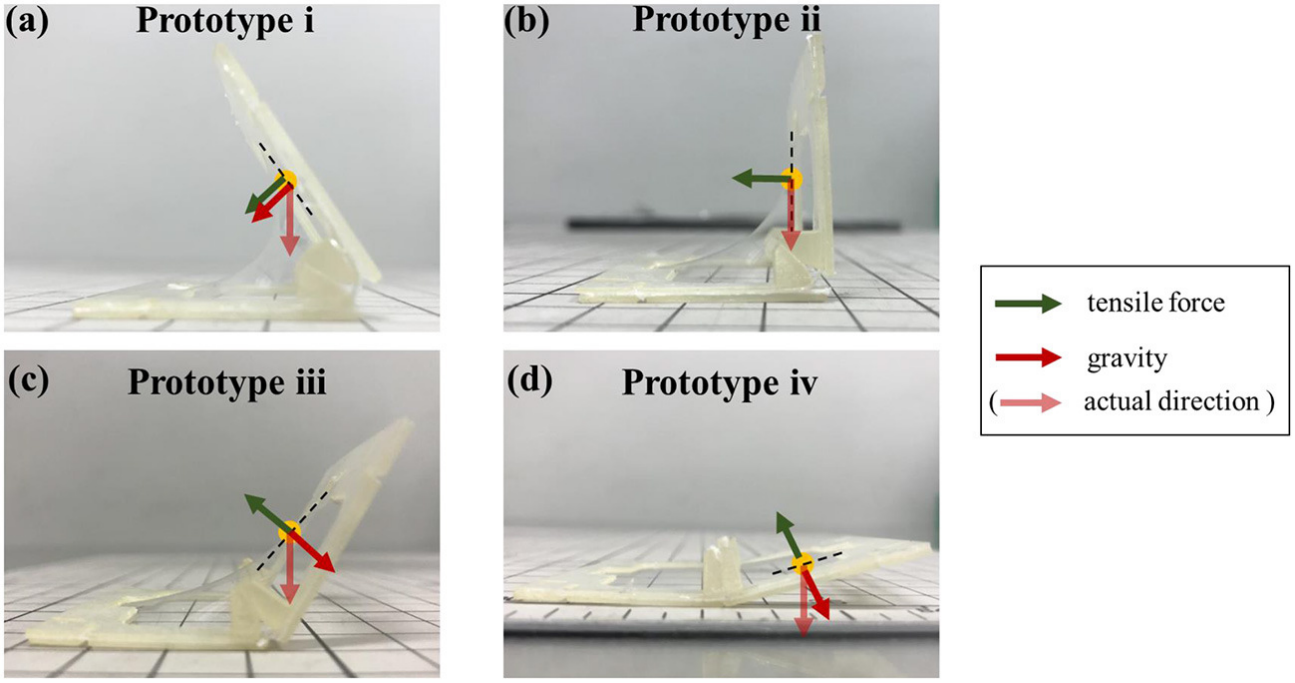
Tension and gravity acting on each prototype. **(a)** In prototype i, the direction of the tensile force to self-fold and the direction of the gravity faces in the same direction. **(b)** In prototype ii, the directions of tension and gravity do not oppose each other. **(c,d)** As in the prototype iii and iv, when the target angle θ_*t*_ is obtuse, the directions of tension and gravity oppose each other.

Figure 10 shows the relationship between the tension and gravity acting in the direction perpendicular to the tangent at a certain point of the elastomer. In Prototype i, when the target angle θ_*t*_ is acute, the direction of the tensile force to self-fold and the direction of the gravity faces in the same direction (Figure 10a). Therefore, the folding angle θ_*m*_ is stable near the target angle, because folding is limited by the hinge. Even if the design angle θ_*t*_ is a right angle as in Prototype ii, the directions of tension and gravity do not oppose each other (Figure 10b). The self-folded angle with Prototype ii also becomes stable as Prototype i. The small difference occurred in the acute angle, because the 3D structure was slightly curved by thinning the frame to reduce weight of the frame.

On the other hand, when the target angle θ_*t*_ is obtuse, as in the prototype iii and iv, the difference occurs in the opening direction, because the gravity pushes the self-folded structure to the ground direction (Figures 10c,d). The reason why the standard deviation is large and the bending angle is not stable is also considered to be the opposite direction of tension and gravity.

We found that to designing the folding angle is feasible with the effect of the gravity. By further reducing the weight of the frame by changing the material, the influence of gravity will be reduced.

### Displacement Measurement of the Self-Assembled Actuator Under DC Voltage Application

#### Experimental Condition

When voltage is applied to the self-assembled actuator, the elastomer extends to increase the folding angle. We measured the angular displacement ϕ_*m*_ with the application of different voltage. Each prototype was initially given a voltage of 1 kV, and then voltage was increased by 1 kV. Since the actuator was broken with 6 kV of voltage, we set the maximum voltage 5 kV. Experiments for each prototype was performed four times. In each trial, voltage was applied for at least 8 s to observe the response of the actuators. We used image analysis software (Kinovea) to measure the angle.

### Results and Discussions

**Figure 11:**
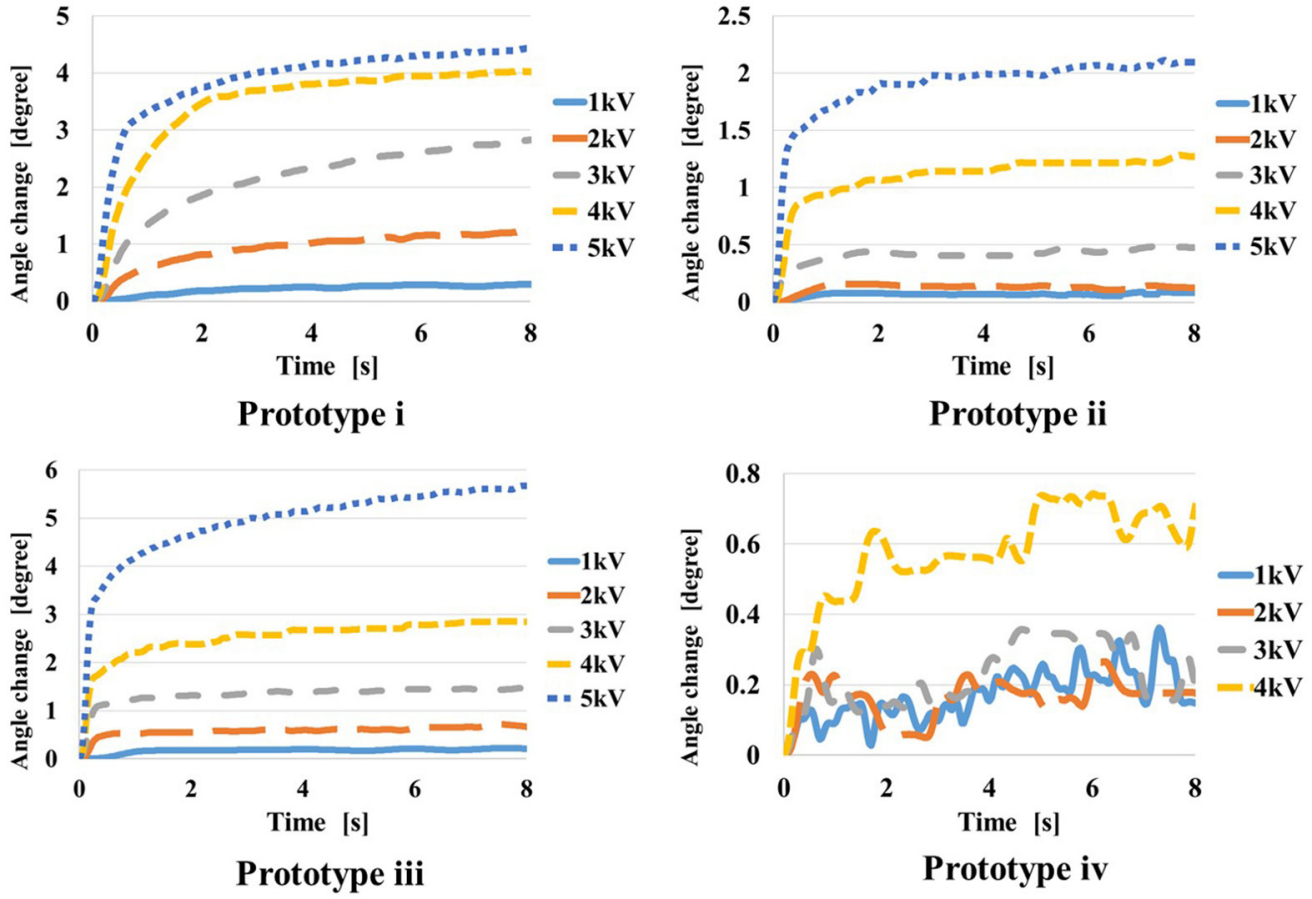
Result of applying DC voltage to each prototype.

Figure 11 shows the relationship between the applied voltage and the angular displacement for the prototypes i~iv. One of the results out of four trials are shown. The reference angle for starting measurement is the angle at which the three-dimensional structure is formed by self-folding and the angle is fixed. In this experiment, it was considered that the angle change was sufficiently completed after 8 s, so the angle after 8 s from the start of voltage application was defined as the value when the actuation end. The greater voltage was applied, the greater folding angle was obtained. The maximum angle around 6° was obtained by applying 5 kV of voltage to prototype iii. In prototype iv, since it was broken when 5 kV was applied, the data with applying 1~4 kV of voltage was plotted in the graph.

**Figure 12:**
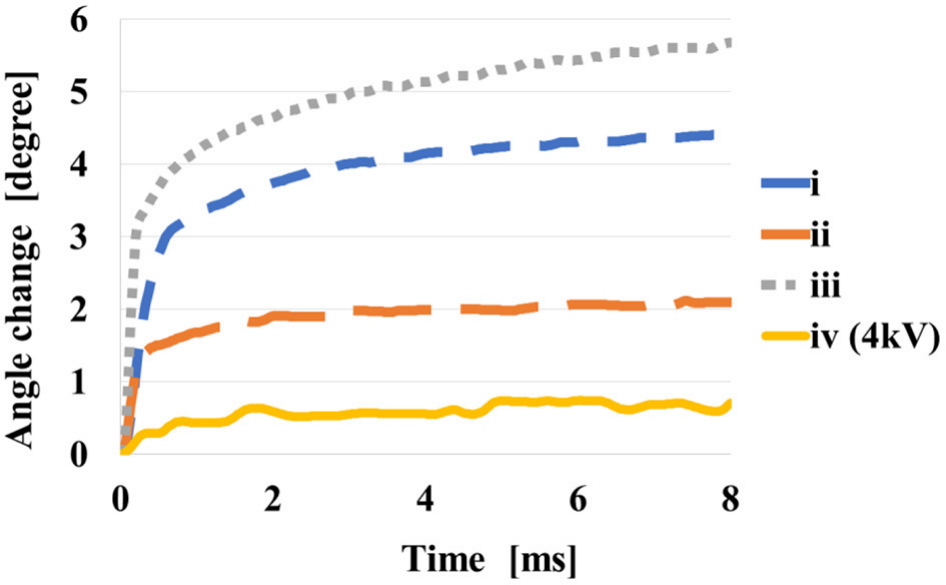
Comparison of four prototypes at the applying voltage of 5 kV.

Figure 12 shows the temporal response of angular displacements when 5 kV is applied to prototypes i to iv. Table 2 shows the results of the angular displacements after 8 s from the voltage application. For each prototype, 4 trials were performed and the average and the variance are shown. In each trial, small variance is found, however expected actuation is obtained by the application of voltage. The angular displacement increases in the order of prototype iii, i, ii.

**Table 2:** Measurement results of angular displacement by applying DC voltage.

		**i (60°)**	**ii (90°)**	**iii (120°)**
Angular displacement (degree)	Trial 1	4.44	2.09	5.67
	Trial 2	3.11	3.02	5.12
	Trial 3	4.43	2.15	4.89
	Trial 4	3.97	2.09	5.67
	Average	3.98	2.54	5.17
	Variance	0.29	0.18	0.08

When voltage is applied, the elastomer expands, so the balance between gravity and tension changes, and the contribution of gravity increases. This change in balance is considered to be greater when tension and gravity work in opposite directions, that is, when the folding angle is obtuse. Because the direction of opening coincides with the direction of gravity. The reason why the change in the prototype iv is small is thought to be because it was greatly affected by gravity at the time of self-folding, and the change in force balance by voltage applying was not so large. It can be considered that the structure of the prototype iii is most affected by this effect. From this experiment, we consider the shape of the prototype iii is suit to obtain larger displacement as the actuator.

In addition, the response relaxations are found in Figures 9, 10. These relaxations are considered to be caused by the viscosity of the elastomer. The acrylic elastomer (VHB 4905-J) used in this experiment is known to have the characteristics of higher viscosity and lower elasticity than silicon elastomer. Michel et al., show that the viscosity in acrylic elastomers can be minimized by pre-straining those (Michel et al., 2009). According to an experiment introduced by Shigemune et al., when 2 kV DC voltage was applied to an acrylic elastomer with 300% pre-strain, it took about 3,000 s to relax the strain (Shigemune et al., 2018). In our proposed mechanism, the elastomer is fixed with 300% pre-strain, so the viscosity can be kept small. Furthermore, since the motion direction of the self-assembled actuator is amplified by the effect of the gravity, the relaxation time is shorter than the previous study. Therefore, our proposed mechanism has the structure that can enhance the response of the elastomer by voltage application. In this experiment, the angular displacement in 8 s from the start of voltage application was recorded. Although the angular displacement gradually increases after 8 s, the figures properly show the response of the actuator to voltage application.

### Displacement Measurement of the Self-Assembled Actuator Under AC Voltage Application

#### Experimental Condition

We conducted an experiment to investigate the response characteristics of the self-assembled actuators. We applied rectangular waves of 15 patterns of frequencies (0.1, 0.3, 0.5, 1.0, 2.0, 3.0, 4.0, 6.0, 8.0, 10.0, 15.0, 20.0, 30.0, 50.0, 100 Hz) to prototypes i and iii for 20 s. We selected prototypes i and iii, since the prototypes showed better performance with the DC voltage application. Difference between the maximum value and the minimum value of the angle between 5 and 20 s is plotted. This experiment was also performed four times for each prototype.

### Results and Discussions

**Figure 13:**
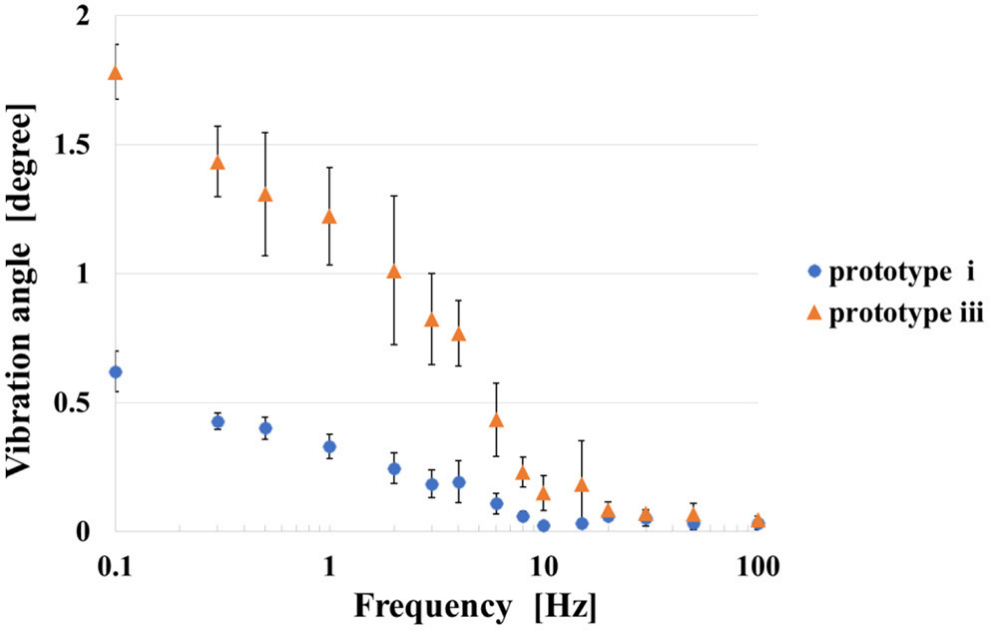
Result of applying 5 kV AC voltage to prototype (i) and (iii).

Figure 13 shows the results of the experiment. The plot in the figure represents the average of four trials, and the error bar represents the standard deviation of the four data. The angular displacement decreases as increasing the applied frequency. According to the result, the self-assembled actuator could respond to the rectangular wave with frequency up to 10 Hz. The standard deviation increases around 1 to 10 Hz. The prototype iii, which has the structure in which tension and gravity work in opposite directions, has a larger angular displacement than the prototype i when a voltage of the same frequency is applied.

### Force Measurement of the Self-Assembled Actuator

#### Experimental Condition

**Figure 14:**
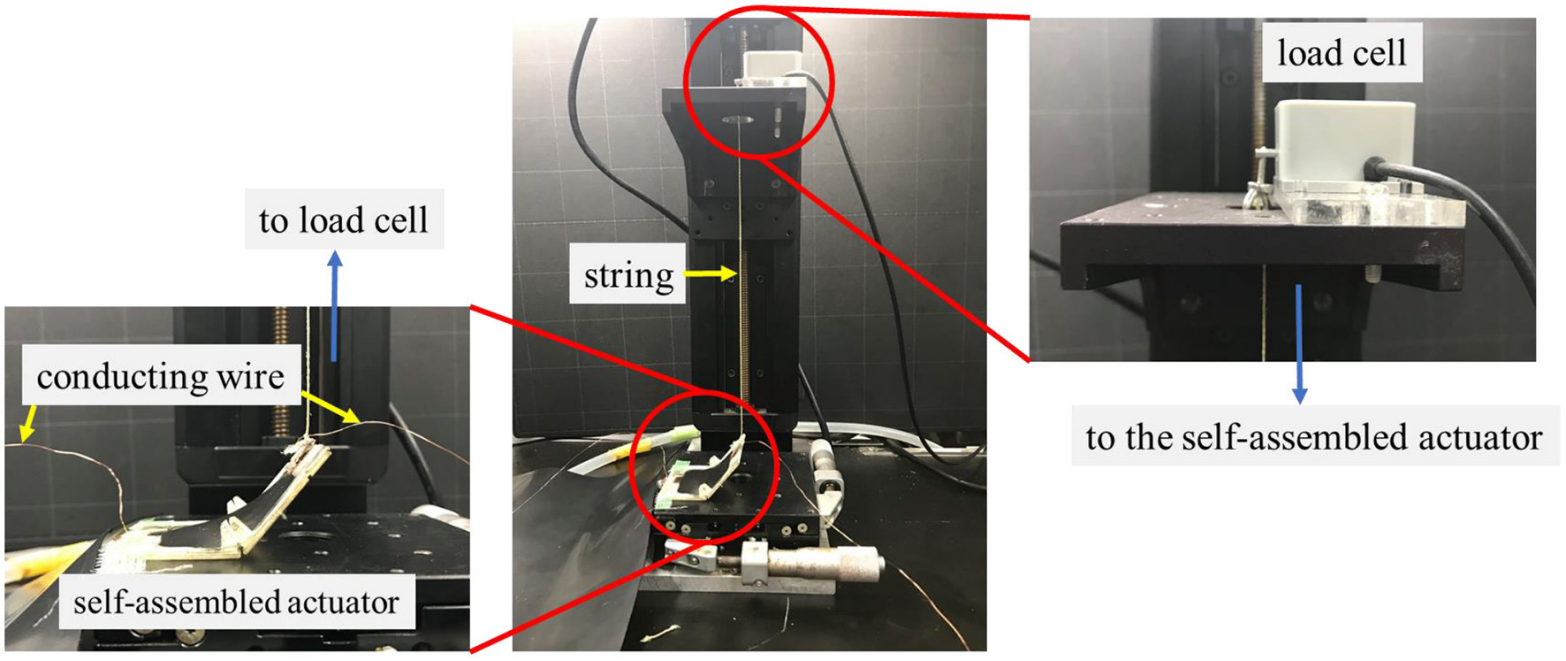
The experimental environment for measuring the force generated by the self-assembled actuator. When a voltage is applied to the actuator, the string connected to the load cell is pulled downward. That force is recorded by the load cell.

We measured the force generated by the self-assembled actuator. The experimental environment is shown in Figure 14. We fixed the self-assembled actuator to the base, and connected the load cell at the tip of the actuator with a string. At this time, the string was set to remain taut. When a voltage is applied to the actuator, the actuator is driven toward the opening direction to pull the string. In this experiment, Prototype iii that showed the best performance in Experiments 4.2 and 4.3 was used. We measured the force generated with applying voltage of 5 kV for four times.

### Results and Discussions

Table 3 shows the results of the experiment. The average force of the four actuators was 0.66 N. Since the average weight of the four prototypes used in the experiment was 3.30 g, the gravity applied to the entire actuator is approximately 0.0033 [kg] ×9.8 [m/s] = 0.032 [N]. Therefore, the effect of gravity is negligible compared to the force generated by the actuators. By considering the generated force, the actuator mechanism has great potential to be applied for various applications including a gripper which is demonstrated at later section.

**Table 3:** Measurement results of the force generated by the actuator.

	**Trial 1**	**Trial 2**	**Trial 3**	**Trial 4**	**Average**
Driving force (N)	0.63	0.65	0.62	0.76	0.66

## Application For a Gripper

### Design and Concept

Based on the experimental results, we attempted to apply the self-assembled actuator for a gripper. In order to achieve a large gripper operation by applying voltage, we select the self-folded angle 120° referring to the results described above.

**Figure 15:**
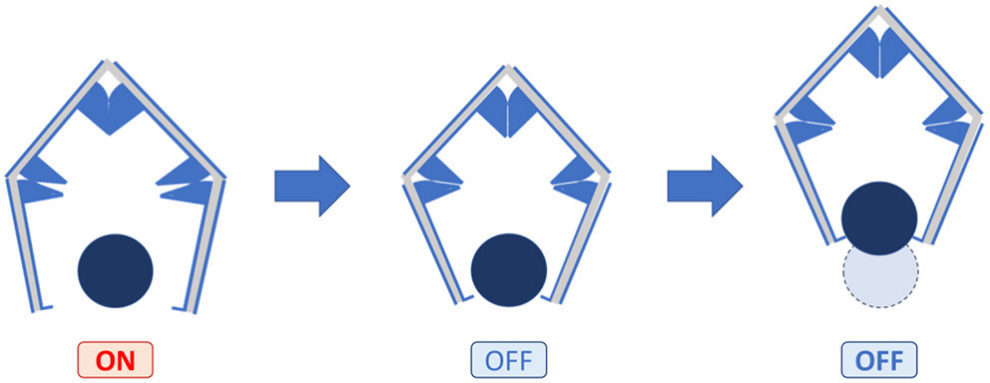
Concept of self-folded gripper. The left and right joints open when a voltage is applied, and close when the voltage is removed.

Figure 15 shows the concept of a self-assembled gripper. The gripper has a structure, in which three joints are connected in series. Among the joints, an elastomer with electrodes is fixed at two places on both sides, and when voltage is applied, the joints are actuated in the direction of opening. The gripper achieves the gripping of an object by switching the voltage. The gripper opens while switching ON, and closes while switching OFF. The gripper keeps closing while switching OFF, therefore the device does not consume energy to grab an object.

**Figure 16:**
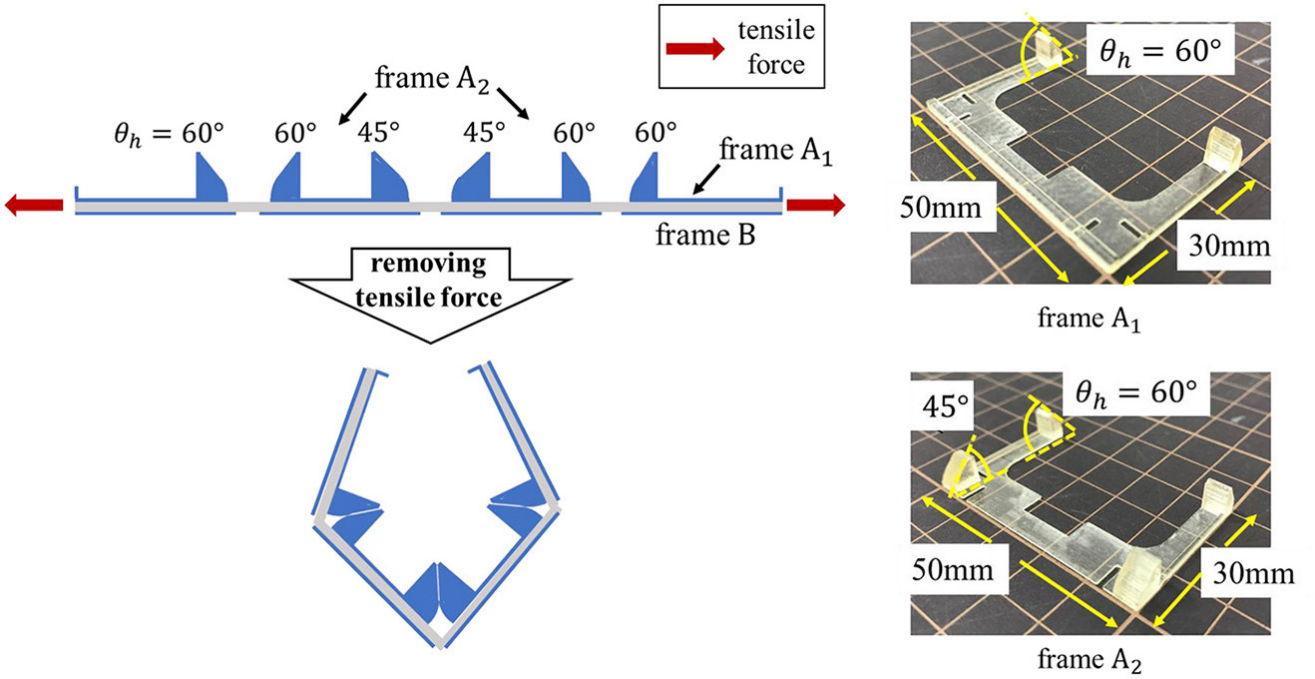
Blueprint of the gripper and appearance of frames *A*_1_ and *A*_2_.

Figure 16 shows the blueprint of the gripper and the appearance of the frame used. Based on the results of Experiments 4.2 and 4.3, the hinge angle was designed as shown in the figure. Frame *A*_1_ has a claw hook at tips to make it easier to grab.

**Figure 17:**
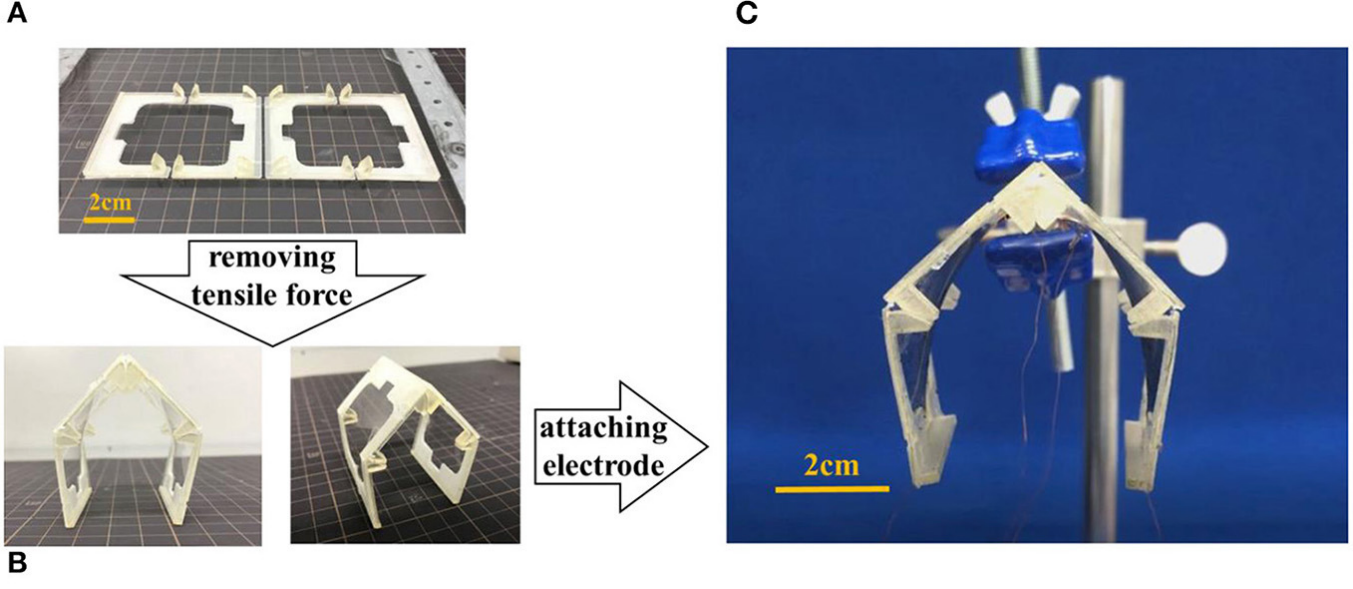
The structure of the gripper. The center joint was designed to be 90°, and the left and right joints were designed to be 120°. **(A)** Frames are pasted on an elastomer given pre-strain of 300%. **(B)** When the elastomer outside the frame is removed, the gripper structure is formed. **(C)** Appearance of the gripper.

Figure 17 shows the process of making a gripper. Figure 17A shows a state in which frames are pasted on an elastomer given pre-strain of 300% as shown in Figure 16. When the elastomer outside the frame in the state (a) is removed, the gripper structure is formed (Figure 17B). Finally, the CNT electrode is attached to the elastomer to complete the gripper (Figure 17C).

The gripper can hold various objects by changing the design of the frame according to the shape of the object. Since the gripper has a flexible joint, it can hold the object even if its size slightly changes.

### Performance

**Figure 18:**
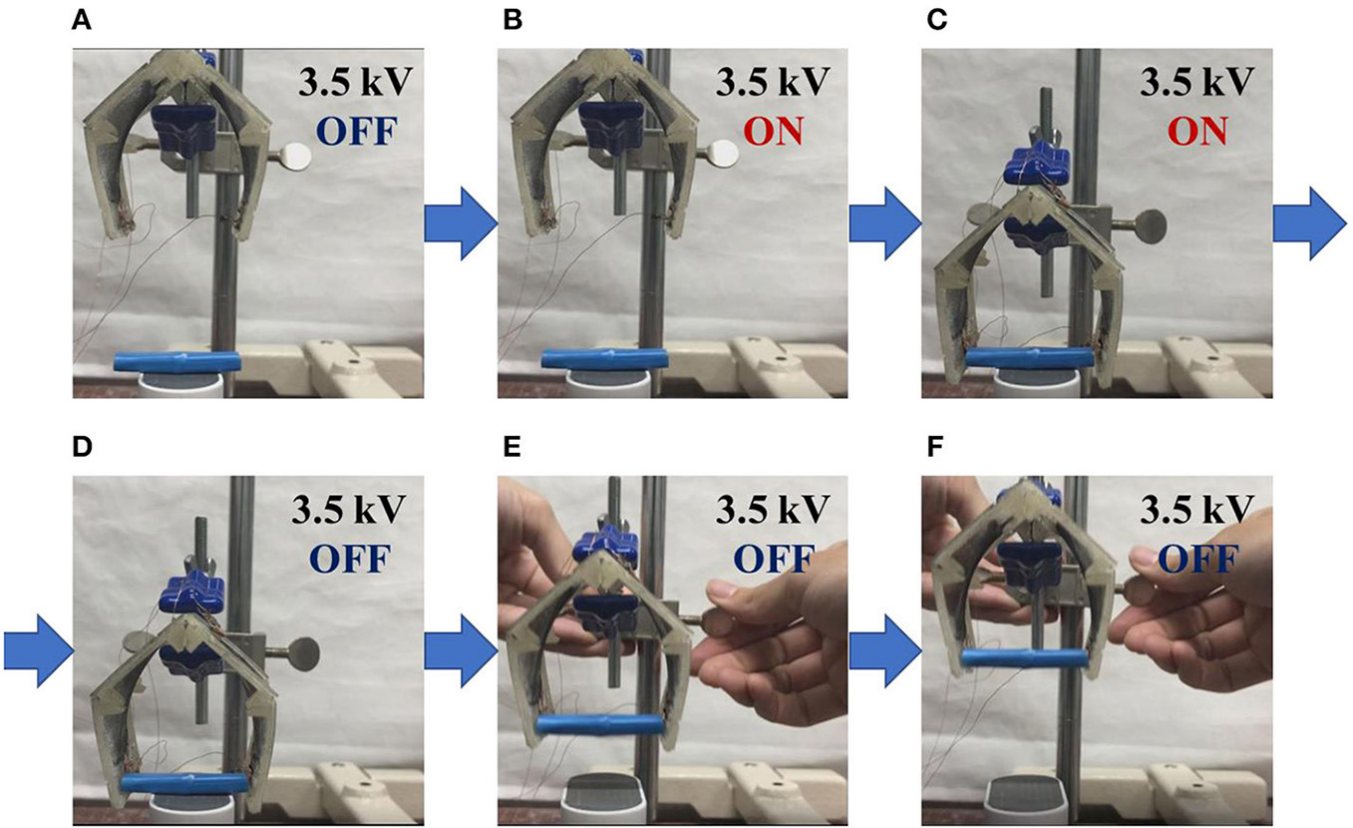
Demonstration to grip an object. The gripper achieves in gripping the object by switching the voltage. **(A)** Initially, the gripper is closed. **(B)** When voltage is applied, the gripper opens. **(C)** Move the open gripper closer to the object. **(D)** When the voltage is turned off, the gripper grips the object. **(E,F)** The object can be lifted by moving the gripper.

We verified the performance of the gripper by letting grab an object. The gripper grabs an object, and manually transfers to another position. Figure 18 shows the procedures of the attempt. In this experiment, an object with a length of 47.0 mm and a weight of 0.65 g was employed. In the state of (a), no voltage was applied to the electrode and the gripper was closed. When a voltage was applied, the gripper was in the open state (b). The gripper approaches to the object (c), then the voltage is turned off to grasp the object (d). The gripper successfully grasps the object with the length was 36.7 mm and weight up to 3.28 g.

## Conclusion

In this research, we proposed a self-assembled actuator using an elastomeric material and a rigid frame. We evaluated accuracy of the self-folding actuators, and carried out an experiment to investigate the performance of the self-assembled actuator by applying DC and AC voltage. We attempted to apply the actuator for a self-assembled gripper, and achieved to grab an object with the length of 36.7 mm and the weight of 3.28 g. The 3D structure formation by self-folding was limited by the hinge when the design angle was obtuse, so the standard deviation of data was small. On the other hand, when the design angle was acute, the folding angle was determined by the balance between tension and gravity, so the standard deviation of data was relatively large. When DC voltage was applied, the folding angle suitable for the largest displacement was 120°. When AC voltage was applied, the prototypes were confirmed to respond at frequencies below 10 Hz. In the future work, we try to assemble much complex structures using multiple hinges. Complex self-assembled actuators will realize flexible locomotion by utilizing its flexible joints.

## Data Availability Statement

The datasets generated for this study are available on request to the corresponding author.

## Author Contributions

All authors made an intellectual contribution to this paper and acknowledged the publication. In particular, NH designed and fabricated the devices, designed the experimental setup, analyzed the data, contributed to data interpretation, and wrote the paper. HSh designed the concept of the project, contributed to data interpretation, and wrote the paper. AM designed the experimental setup and wrote the paper. HSa and SM provided advice to fundamentals of the research and wrote the paper.

### Conflict of Interest

The authors declare that the research was conducted in the absence of any commercial or financial relationships that could be construed as a potential conflict of interest.

## Supplementary Material

Supplementary Video This video shows the process of fabricating a self-folding prototype shown in Figure 5.
